# Structure and Functionality of Fermented Faba Bean: Influence of Particle Size and *Rhizopus* spp.

**DOI:** 10.3390/foods14234105

**Published:** 2025-11-29

**Authors:** Deepa Agarwal, Priyanka Kharangarh, Pengfei (Alfie) Hao, Mark I. Bradbury, Pankaj Maharjan, Yakindra P. Timilsena, Cassandra K. Walker, Monika S. Doblin, Roman Buckow

**Affiliations:** 1 La Trobe Institute for Sustainable Agriculture and Food, La Trobe University, Bundoora, VIC 3086, Australiaa.hao@latrobe.edu.au (P.H.); m.doblin@latrobe.edu.au (M.S.D.); 2 School of Agriculture, Biomedicine and Environment, Department of Ecological, Plant and Animal Science, La Trobe University, Bundoora, VIC 3086, Australia; 3 School of Allied Health, Human Services and Sport, La Trobe University, Bundoora, VIC 3086, Australia; 4 Agriculture Victoria Research, Horsham SmartFarm, Horsham, VIC 3400, Australia; pankaj.maharjan@agriculture.vic.gov.au (P.M.);; 5 School of Applied Systems Biology, La Trobe University, Bundoora, VIC 3086, Australia

**Keywords:** Vicia faba, particle size, *R. oligosporus*, *R. oryzae*, solid-state fermentation

## Abstract

This study investigated the influence of particle size and *Rhizopus* species on the fermentability, structure, and functionality of faba bean (*Vicia faba* L.) during controlled solid-state fermentation. Split seeds, coarse particles, and 1000–2000 µm fractions were fermented with either *R. oryzae* or *R. oligosporus*. Analyses included compositional profiling, SDS-PAGE, FTIR, DPPH antioxidant activity, phytic acid quantification, and rheological yield stress measurements. Particle size strongly affected mycelial growth and matrix structure: coarse particles supported more uniform mycelial networks, particularly with *R. oryzae*. After 48 h of fermentation, total protein and phytic acid contents remained largely unchanged; however, SDS-PAGE and FTIR results indicated proteolysis and alterations in secondary structure, accompanied by higher antioxidant activity. Rheological data showed significant species–particle size interactions influencing yield stress, with *R. oligosporus*-fermented samples exhibiting higher yield stress than those fermented with *R. oryzae*. Overall, these findings demonstrate that optimising particle size and fungal strain combinations can enhance the structural and functional characteristics of fermented faba bean.

## 1. Introduction

Faba bean (*Vicia faba* L.), also known as broad, horse, or field bean, is one of the earliest legumes to be domesticated because of its nutritional profile and agronomic benefits [1]. There are two main types: *V. faba* major (broad bean) characterised by large seeds and are predominantly grown in southern Europe for human consumption, and *V. faba minor* (field bean) with smaller seeds, used for both food and feed [2]. Globally, faba bean is valued for its high contents of protein (~30%), carbohydrates (44–47%), total dietary fibre (8%), essential minerals, and bioactive compounds [3,4]. Beyond nutrition, faba bean supports sustainable agriculture through nitrogen fixation [5]. However, its broader utilisation is constrained by antinutritional components such as pyrimidine beta-glycosides vicine and convicine, saponins, protease inhibitors, and phytic acid, as well as undesirable flavours and textural limitations [6,7]. Vicine and convicine pose health risks to individuals with glucose-6-phosphate dehydrogenase (G6PD) deficiency, potentially triggering a condition known as favism [8]. Lipoxygenase-mediated oxidation of unsaturated fatty acids generates volatiles linked with ‘beany’ flavour, and thus, consumer acceptance issues [9]. Conventional processing methods such as soaking, heating, and germination reduce some of these limitations [4,10,11], yet strategies that enhance both nutrition and sensory quality remain needed.

Fermentation offers a promising approach. By transforming complex substrates into simpler compounds, fermentation improves food safety, stability, digestibility, and sensory appeal, while reducing antinutritional factors [12]. In legumes, fermentation increases protein digestibility, lowers phytic acid, produces bioactive metabolites, and degrades oligosaccharides associated with flatulence [13,14]. These benefits align with growing demand for sustainable and allergen-free foods.

Among microorganisms employed in legume fermentation, *Rhizopus oligosporus* is a common starter culture for tempeh. It grows optimally at 30–42 °C and produces a broad range of exogenous carbohydrate-active enzymes (CAZymes), alongside proteolytic and lipolytic enzymes [15]. These enzymes facilitate the breakdown of phytic acid, oligosaccharides, and proteins into simpler compounds, improving texture, flavour, and nutritional bioavailability [16,17]. Its metabolism also produces bioactive compounds, including antioxidants, antimicrobials, organic acids, and vitamins, improving nutritional quality and safety [18]. Similarly, *Rhizopus oryzae*, another filamentous fungus widely employed in Asian fermented foods, exhibits broad pH (4–9) tolerance, can grow at up to 40 °C, and shows industrial versatility [19]. In contrast to *R. oligosporus*, *Rhizopus oryzae* fermentation contributes distinct sensory properties such as mild sour, bitter, and sweet flavours, along with a soft and moist texture. It also improves antioxidant activity, and bioactive metabolite profiles in legumes such as chickpea [20,21]. The proven role of *Rhizopus* spp. in improving nutritional and functional qualities positions them as promising candidates for faba bean fermentation.

Processing conditions strongly impact fermentation outcomes. Pre-treatments such as soaking, boiling, dehulling, and germination can reduce antinutritional factors and improve nutrient accessibility [22]. Beyond these, substrate particle size is emerging as a critical factor shaping fermentation efficiency and product functionality. Smaller substrate particles increase surface area for microbial access, whereas excessively fine particles promote aggregation and restrict oxygen diffusion. On the other hand, larger particles enhance heat and mass transfer but provide comparatively less surface area for colonisation [15]. Fermentation time and temperature further modulate flavour development, antioxidant activity, and nutrient retention [14]. Optimising these parameters is therefore critical to achieving desirable texture, nutritional quality, and sensory attributes in faba bean products.

Despite growing interest in plant-based proteins, faba bean fermentation remains understudied relative to soybeans, chickpeas, and peas. Research has addressed some pre-processing methods to reduce antinutrients, yet little is known about how fermentation, particularly with *Rhizopus* spp., interacts with particle size to influence protein structure, rheological behaviour, antioxidant activity, and antinutrient reduction. Substrate particle size is known to strongly impact microbial growth and nutrient accessibility, but its role in shaping the structural and functional quality of fermented faba bean products is poorly defined, particularly in relation to process uniformity.

This study investigates the impact of particle size and different *Rhizopus* species on nutritional and textural properties of fermented faba bean. We evaluate the effects on protein content, secondary structure, rheology (yield stress), antioxidant capacity (DPPH), and antinutritional content (phytic acid). The novelty lies in elucidating how particle size modulates fermentation efficiency and quality outcomes, providing new insights to enhance faba bean fermentation for diverse food applications.

## 2. Materials and Methods

### 2.1. Material

Split faba beans were sourced from Essantis (Melbourne, Australia). Commercial starter cultures of *Rhizopus oligosporus* were obtained from Nourishme Organics (Melbourne, Australia) and *Rhizopus oryzae* from Kensho via Naturita (Amsterdam, The Netherlands). Phytic acid analysis was performed using the Megazyme Phytic Acid Assay Kit (Neogen, Ireland). Other analytical reagents, including ascorbic acid, sulfuric acid, ammonium molybdate, trichloroacetic acid, hydrochloric acid (37%), and sodium hydroxide, were purchased from Merck Life Science (Melbourne, Australia). Sodium phosphate monobasic, sodium phosphate dibasic, potassium sulphate, and 2-mercaptoethanol were also obtained from Merck Life Science (Australia). Precision Plus Protein™ Dual Color Standards and 4× Laemmli sample buffer were sourced from Bio-Rad, and Coomassie Brilliant Blue G-250 from Thermo Fisher Scientific. DPPH (2,2-diphenyl-1-picrylhydrazyl), methanol, Tris-HCl, glacial acetic acid, and potato starch were obtained from ChemSupply (Adelaide, Australia).

### 2.2. Substrate Preparation and Preprocessing

Faba beans of three different particle sizes (split, coarse, and 1000–2000 µm) were prepared according to Table 1 and used as fermentation substrates. A total of 1000 g of material was prepared for each fermentation trial. For each replicate, 60 g of split, coarse, or 1000–2000 µm faba bean sample was placed in a separate polypropylene box (144 mm L × 60 mm H × 105 mm W) and sterilised by autoclaving at 121 °C for 15 min. Milli-Q water was sterilised separately under the same conditions. After cooling to room temperature (RT), the autoclaved samples were soaked in Milli-Q water at substrate-to-water ratios of 1:1 (6 h), 1:1 (6 h), and 5:4 (3 h) for split, coarse, and 1000–2000 µm beans, respectively, to ensure a consistent initial moisture content (50 ± 2%) across all samples. After soaking, excess water was drained, and the samples were used for fermentation. Moisture content of the pre-fermented beans was determined in triplicate using the oven-drying method according to AOAC Official Method 934.01 [23].

### 2.3. Solid-State Fermentation Process

An inoculum of *R. oligosporus* (initial concentration 0.96 × 10^9^ spores/mL) was prepared according to the manufacturer’s instructions by mixing the starter culture directly from the pack with potato starch at a 1:9 ratio. The inoculum was added at a culture-to-substrate ratio of 1:500. The *R. oryzae* inoculum (1.16 × 10^9^ spores/mL) was applied directly from the pack at a culture-to-substrate ratio of 1:200, with no additional potato starch added, as the starter culture already contained rice starch. Initial spore concentrations were confirmed microscopically using a Neubauer haemocytometer and expressed as spores per mL. Fermentation was conducted under static, aerobic conditions in loosely covered sterile containers to permit gas exchange. The process was carried out for 48 h at 25 °C for each particle size with ambient relative humidity maintained at approximately 60–65%, with all treatments performed in triplicate. Preliminary experiments showed that these fermentation conditions resulted in consistent mycelial growth, and substrate colonisation without signs of overgrowth or substrate degradation. No external mixing was applied during fermentation to maintain the integrity of solid-state conditions. Moisture content, pH, rheological measurements, and visual analyses were conducted on fresh samples immediately after 48 h of fermentation. All remaining samples were freeze-dried and milled before further analyses, including compositional assessment, SDS-PAGE, FTIR, antioxidant activity (DPPH), and antinutritional (phytic acid) analysis.

### 2.4. Qualitative Analysis

Qualitative observations, including visual mycelial coverage, texture, colour, and aroma, were recorded for fresh samples straight after 48 h of fermentation. Images of the fermented faba beans were taken from the top, bottom, and cross-sectional views to document surface and internal structural features.

### 2.5. Compositional Analysis

Freeze-dried fermented faba bean samples were ground to a fine powder prior to analysis. Total fat content was determined according to AOAC Official Method 920.39 [24] using a Foss Soxtec 2050 extractor (Foss Pacific Pty Ltd., Hillerød, Denmark). Moisture content was determined following AACC Method 44-15.02 using a LECO TGA 701 (LECO Corporation, Saint Joseph, MI, USA). Ash content was determined according to AACC Method 08-03.01 using the same instrument. Total protein content was measured by dry combustion (950 °C) using a CN Elemental Analyzer (Vario EL Cube, Elementar Analysensysteme GmbH, Hesse, Germany) with argon as the inert carrier gas. Nitrogen content was converted to protein content using a conversion factor of 6.25. Total dietary fibre (DF) was measured using an Ankom Dietary Fibre Analyzer following AOAC Method 991.43, [25] and total carbohydrate (CHO) content was determined colourimetrically using the phenol–sulfuric acid method [26].

### 2.6. SDS-PAGE (Sodium Dodecyl Sulphate-Polyacrylamide Gel Electrophoresis)

Total protein was extracted from freeze-dried, ground faba bean samples using a sample-to-buffer ratio of 1:10 (*w*/*v*). Samples (0.1 g) were extracted in 1 mL of 0.1 M sodium phosphate buffer (pH 7.2) containing 5 g/L potassium sulphate. After brief vortexing, the samples were stirred at 80 °C for 30 min and centrifuged at 20,000× *g* for 30 min at room temperature. Supernatants were transferred to fresh tubes and stored at −20 °C. Protein extracts were diluted 1:4 (*v*/*v*) with Milli-Q water prior to SDS-PAGE. The loading dye was prepared by adding β-mercaptoethanol to Bio-Rad 4× Laemmli Sample Buffer (125 mM Tris-HCl, pH 6.8; 2% *w*/*v* SDS; 10% *v*/*v* glycerol; 0.01% *w*/*v* bromophenol blue) at a 1:9 (*v*/*v*) ratio. Samples were mixed with the loading dye and denatured at 95 °C for 5 min, then cooled to room temperature. Ten microlitre aliquots were loaded onto Bio-Rad (Hercules, CA, USA) stain-free precast gels (4–15%) and electrophoresed at 100 V for 75 min. Precision Plus Protein™ Dual Colour Standards (Bio-Rad) with a molecular weight range of 10–250 kDa were used as markers. Gels were stained with 1 g/L Coomassie Brilliant Blue G-250 in methanol/acetic acid/water (500:100:400, *v*/*v*/*v*) for 30 min and destained overnight in methanol/acetic acid/water (100:100:800, *v*/*v*/*v*). The stained gels were visualised using a Bio-Rad ChemiDoc™ Imaging System. Band intensities were quantified using ImageJ software (v1.54, NIH, Bethesda, MD, USA) by converting gel images to grayscale and measuring pixel intensity profiles for selected protein bands. Results were expressed as relative intensity values normalised to the unfermented control.

### 2.7. Fourier Transform Infrared Spectroscopy (FTIR) Analysis

FTIR analysis of freeze-dried fermented samples was performed using an IRXross FT Spectrophotometer (Shimadzu Corporation, Kyoto, Japan) equipped with a DLATGS (deuterated L-alanine-doped triglycine sulphate) detector and an ATR diamond crystal. Samples were placed directly on the ATR crystal at room temperature. Spectra were recorded using LabSolutions IR software (v1.122) with a spectral range of 3500–500 cm^−1^, a resolution of 4 cm^−1^, and eight co-added scans per sample. Background spectra were recorded under identical conditions and automatically subtracted. The Amide I region (1600–1700 cm^−1^) was baseline-corrected, normalised, and deconvoluted using Gaussian fitting to quantify protein secondary structure components. Spectral data were mean-centred and normalised using Unscrambler X 10.6 (CAMO Software Pvt Ltd., Oslo, Norway). Principal component analysis (PCA) was applied to normalised spectra using vector normalisation to identify spectral differences among fermented samples.

### 2.8. Rheological Analysis

Freshly fermented samples (48 h) were gently crushed with a mortar and pestle to slightly disrupt the structure for loading. Rheological measurements were performed using a stress-controlled rheometer (MCR 302e, Anton Paar, Graz, Austria) equipped with a 25 mm serrated parallel plate (PP25/P2) at 25 ± 1 °C under Peltier temperature control. Approximately 1 g of sample was loaded between plates with a 2 mm gap. Amplitude sweeps were conducted at strain amplitudes ranging from 0.001 to 150% at a constant angular frequency of 10 rad·s^−1^. Yield stress was defined as the stress corresponding to a 5% reduction in the storage modulus (*G*′) relative to the linear viscoelastic region. Data are presented as mean values of triplicate measurements.

### 2.9. Antioxidant Activity

To prepare the sample extracts, 1 g of freeze-dried faba bean sample powder was mixed with 10 mL of 80% methanol and kept on an orbital shaker for 3 h at RT. Subsequently, the samples were centrifuged at 3000× *g* for 30 min, and the supernatant collected. A 1 mL aliquot of 0.1 mM DPPH (2,2-diphenyl-1-picrylhydrazyl) solution was added to 1 mL of sample extract and 1 mL of methanol to prepare test samples and control, respectively. After brief vortex (for 30 s), the mixtures were incubated at RT for 30 min in the dark. The absorbance was then recorded via a UV-visible spectrophotometer (Shimadzu U-1800) at 517 nm, using methanol as a blank. All the samples were tested in triplicate. The antioxidant activity was measured by calculating the percentage of DPPH radical inhibition. The DPPH scavenging capacity was calculated according to Equation (1).

DPPH-scavenging effect (%) = [(*A_cont_* − *A_test_*)/*A_cont_*] × 100 (1)

where *A_cont_* was the absorbance of the control reaction, and *A_test_* was the absorbance of the sample extracts.

### 2.10. Antinutritional Analysis

Phytic acid, a major antinutritional factor in faba beans, was quantified using the Megazyme Phytic Acid Assay Kit with minor modifications. Each fermented sample (0.5 g) was mixed with 10 mL of 0.66 M (2.4%) hydrochloric acid, vortexed for 30 s, and placed at a 45° angle in a shaking incubator (16 h, 300 rpm). After centrifugation at 3200× *g* for 5 min, 1 mL of the supernatant was transferred to a microfuge tube and centrifuged again at 16,000× *g* for 10 min. A 0.5 mL aliquot of the clarified supernatant was neutralised with 0.5 mL of 0.75 M NaOH and vortexed thoroughly. Neutralised samples were enzymatically hydrolysed and dephosphorylated using phytase and alkaline phosphatase according to kit instructions. A phosphorus-free 96-well microplate was prepared with blanks (deionised water), phosphorus standards (0–7.5 µg/mL), and 200 µL of each sample. Samples were tested in triplicate. A 100 µL colour reagent (10% *w*/*v* ascorbic acid in 1 M sulfuric acid and 5% *w*/*v* ammonium molybdate, mixed 5:1) was added, and the plate incubated at 50 °C for 1 h. Absorbance was measured at 655 nm using a SPECTROstar Nano microplate reader (BMG LabTech, Ortenberg, Germany). Phosphorus content (g/100 g) was converted to phytic acid using a factor of 0.28 and expressed on a dry weight basis.

### 2.11. Statistical Analysis

All experiments were performed in triplicate, and data are presented as mean ± standard deviation. Statistical analyses were conducted using one-way analysis of variance (ANOVA) with Tukey’s post hoc test to compare treatment means. Two-way ANOVA was applied to assess interactions between *Rhizopus* species and particle size. Analyses were performed using OriginLab 2024 (OriginLab Corp., Northampton, MA, USA), with significance accepted at *p* < 0.05.

## 3. Results

In this study, three faba bean particle sizes—split (S), coarse (C), and 1000–2000 µm (2K)—were assessed with *R. oryzae*, while only split (S) and coarse (C) fractions were tested with *R. oligosporus*. The 2K fraction was not included for *R. oligosporus* as preliminary trials showed non-uniform colonisation and friable matrices. This likely reflects reduced interparticle voidage and oxygen transfer in finer beds, which limits hyphal penetration and enzyme secretion during SSF. *R. oligosporus*, with shorter mycelia and lower sporulation than *R. oryzae*, is more sensitive to these constraints. Further optimisation such as adjusting bed porosity, inoculum carriers, or coarse–fine blending might be required to achieve consistent fermentation performance.

### 3.1. Morphological and Composition Analysis

**Figure 1:**
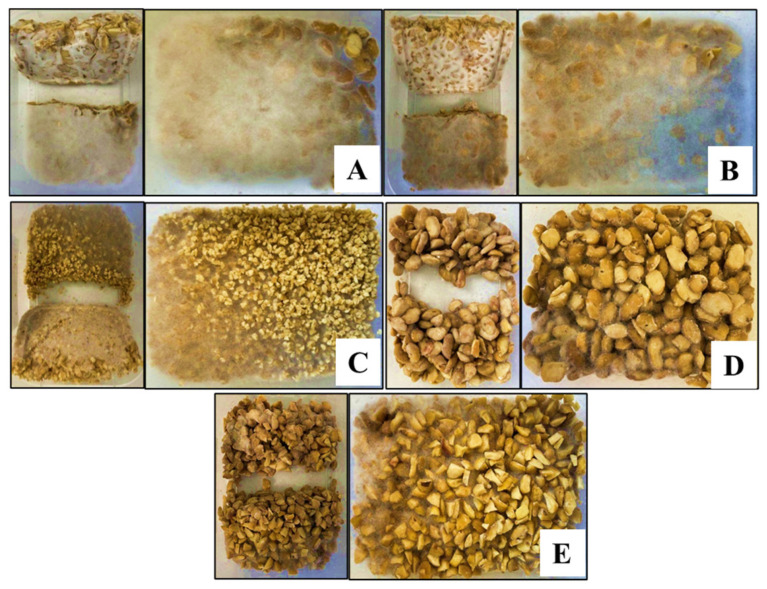
Visual appearance (cross-sectional, top and bottom views) of faba beans after 48 h fermentation, where (**A**) split faba beans, *R. oryzae*; (**B**) coarse faba beans, *R. oryzae*; (**C**) 1000–2000 µm faba beans, *R. oryzae*; (**D**) split faba beans, *R. oligosporus*; (**E**) coarse faba beans, *R. oligosporus*.

Morphological changes during solid-state fermentation of faba bean with *R. oligosporus* and *R. oryzae* were visually assessed to evaluate the impact of particle size and microbial strain after 48 h of fermentation (Figure 1, Table 2). A visible mycelial network developed on both the cross-sectional and exterior surfaces, forming a compact, cake-like structure, particularly in samples inoculated with *R. oryzae*. Split (S), coarse (C), and 1000–2000 µm (2K) faba beans fermented with *R. oryzae* exhibited dense to moderate mycelial growth and a yellow–brown coloration. In contrast, split and coarse samples fermented with *R. oligosporus* showed moderate to slight mycelial coverage and similar yellow–brown hues.

At the start of fermentation (0 h), the moisture content across all samples was 48–50% (Supplementary Figure S1). A slight decrease was observed over time, with final values (48 h) ranging between 41 and 47% (Table 2). Initial pH values ranged from 6.4 to 6.5, increasing to approximately 7.2 to 7.4 after 48 h (Table 2; Supplementary Figure S1). Given the relatively similar pH and moisture contents across treatments, the observed morphological differences are primarily attributed to the effects of substrate characteristics and microbial strains.

Table 2 presents compositional changes in total protein, carbohydrate (CHO), fat, and dietary fibre contents after 48 h of fermentation. The protein content of unfermented split faba bean was 31.3 ± 1.14%. Fermented samples S_Oz, C_Oz, and 2K_Oz contained 32.54 ± 0.19%, 31.9 ± 0.66%, and 31.71 ± 0.42% protein, respectively. Comparing the influence of microbial strains, S_Og and S_Oz showed comparable protein levels (32.06 ± 0.70% and 32.54 ± 0.19%), while C_Og and C_Oz showed minor differences (31.23 ± 0.25% vs. 31.9 ± 0.66%), which were not statistically significant (*p* > 0.05). Although minor variations were observed in total CHO and fat contents, no statistically significant differences were found (*p* > 0.05).

### 3.2. Protein Analysis

**Figure 2:**
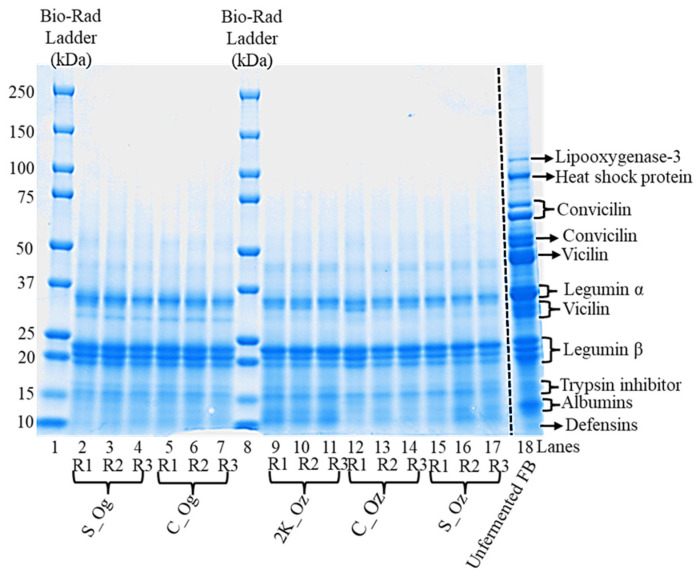
The SDS-PAGE profile of the fermented faba bean at different particle sizes and microorganisms after 48 h of fermentation. Faba bean split (S), coarse (C), and 1000–2000 µm (2K) inoculated with *R. oryzae*, whereas split (S) and coarse (C) inoculated with *R. oligosporus*. Lane 1 and 8: protein molecular weight marker (10–250 kDa); Lane 18: unfermented faba bean (FB) control sample. R1, R2, and R3 within each fermented sample indicate the independent replicates.

SDS-PAGE was used to examine differences in the protein profiles of faba bean before and after fermentation (Figure 2). The unfermented faba bean sample (Figure 2, lane 18) exhibited a broad range of strong, discrete protein bands spanning 10–110 kDa. High-molecular-weight proteins (~95–110 kDa) were observed, likely corresponding to lipoxygenase-3 and heat shock proteins [27]. Prominent bands above ~60 kDa were attributed to conviciline (~65–70 kDa), with a strong protein band at ~50–55 kDa [28]. Protein bands at ~43–48 kDa and ~30–34 kDa were associated with 7S vicilin, a trimeric globulin. Additional bands at ~34–36 kDa and ~20–25 kDa likely corresponded to 11S legumin α and β subunits, respectively [27,29]. Bands below 15 kDa were attributed to minor storage proteins such as albumin, defensins, and trypsin inhibitors [28].

Fermented samples (Figure 2, lanes 2–17) showed similar overall profiles but with notable differences in band intensity and molecular distribution compared with unfermented faba bean. Most bands shifted towards lower molecular weights (<48 kDa), and high-molecular-weight bands (lipoxygenase-3, heat shock proteins, and conviciline) diminished or disappeared. Vicilin-associated bands (~43–48 kDa, ~30–34 kDa) were present but of lower intensity. Densitometric analysis of the vicilin band across all lanes showed more than 40–50% reduction in its intensity, while legumin α and β subunits (~20–32 kDa) remained similar and intensified by up to 50%, respectively, in the fermented samples irrespective of particle size or strain. Bands below 15 kDa were consistently detected across all fermented samples. Minor strain-dependent variations were evident as follows: split and coarse faba beans fermented with *R. oligosporus* (lanes 2–7) exhibited vicilin bands at approximately 30 kDa with intensities exceeding those of other samples by more than 10%. In contrast, samples fermented with *R. oryzae* (lanes 9–17) displayed more pronounced vicilin bands in the 43–48 kDa region, also with >10% higher intensity. Despite these differences, densitometric analysis confirmed an overall reduction of more than 30% in the intensities of these protein bands compared to the unfermented controls. These observations suggest strain-dependent proteolytic activity and protein degradation patterns during fermentation.

### 3.3. FTIR

**Figure 3:**
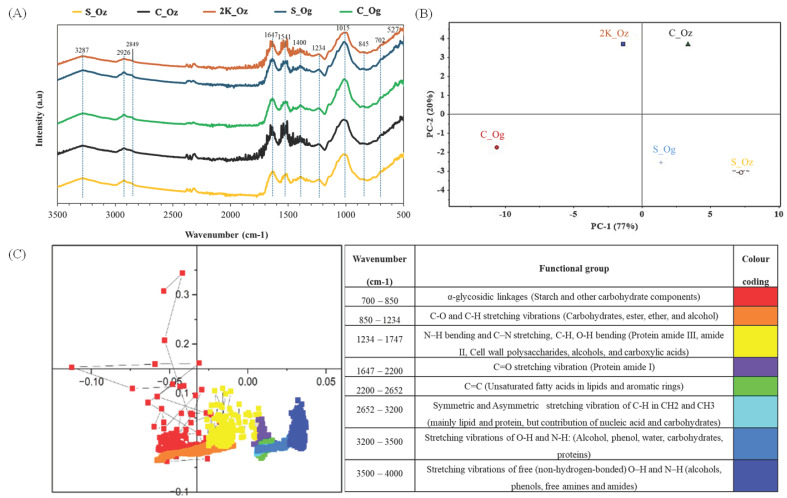
(**A**) The FTIR spectra (3500–500 cm^−1^) of the fermented faba bean split, coarse, and 1000–2000 µm faba beans, inoculated with *R. oryzae* (Oz) or *R. oligosporus* (Og) after 48 h of fermentation. (**B**) The principal component analysis (PCA) plot of average FTIR spectra of each fermented sample. (**C**) PCA loading plot, where different colours (**left**) indicate distinct functional groups correlated with specific wavenumbers (**right**).

FTIR spectra of fermented faba bean samples were obtained to assess the effects of fermentation on carbohydrate fingerprint regions and protein secondary structure (Figure 3A). For reference, FTIR spectra of unfermented faba bean are presented in Supplementary Figure S2. Characteristic absorption bands (3500–500 cm^−1^) corresponding to various functional groups are summarised in Figure 3C. A broad band at ~3287 cm^−1^ was attributed to O-H and N–H stretching vibrations, primarily from water and hydroxyl groups in carbohydrates [30]. Peaks at ~2926–2849 cm^−1^ corresponded to asymmetric C–H stretching in CH_2_ groups, mainly associated with lipids but also present in carbohydrates and proteins [31]. The absorption peak at 1647 cm^−1^ corresponds to the protein amide I band, dominated by C=O stretching (∼80%), and is commonly used to assess protein secondary structure, while the protein amide II band at 1541 cm^−1^ reflects N-H bending and C-N stretching [32]. The 1400 cm^−1^ peak likely corresponds to COO^−^ symmetric stretching in carboxylic acids or CH_3_ bending in proteins [33]. The protein amide III band is linked to the 1234 cm^−1^ observed peak [34]. The region between 1234 and 1015 cm^−1^ corresponds to strong C-O stretching vibrations from C-OH groups in carbohydrates and polysaccharides, while peaks from ~845–702 cm^−1^ are likely associated with α-glycosidic linkages in starch and other carbohydrates [30].

Deconvolution of the amide I region showed a consistent reduction in ordered protein structures following fermentation. In unfermented faba bean, β-sheet and α-helix contents were 5.16% and 1.91%, respectively, whereas in fermented samples these values decreased to 2.61–3.47% for β-sheets and 0.40–1.14% for α-helices (Table 3). Concurrently, unordered and β-turn structures showed moderate increases, indicating partial unfolding and structural rearrangement of proteins. The greatest reduction in α-helical content was observed in C_Og (0.40%) and 2K_Oz (0.73%), suggesting enhanced protein unfolding and enzymatic accessibility in these samples.

Principal component analysis (PCA; Figure 3B) was used to statistically evaluate spectral variation among samples. PC1 and PC2 accounted for 77% and 20% of the total variance, respectively. The loading plot (Figure 3C) identified the wavenumbers contributing to differentiation. Samples 2K_Oz and C_Og were located on the negative side of PC1, while C_Oz, S_Og, and S_Oz clustered on the positive side. This separation indicates that particle size significantly influenced spectral features, particularly protein secondary structure (see Discussion). Microbial strain also affected spectral characteristics: S_Oz and S_Og clustered in the PC1^+^/PC2^−^ quadrant, indicating structural similarities, whereas C_Oz and C_Og occupied distinct PCA regions, reflecting strain-specific effects on protein structure. Deconvolution of the protein amide I region showed reduced β-sheet and α-helix content across all fermented samples compared with the unfermented control (Table 3). Notably, faba beans fermented with *R. oligosporus* (especially C_Og) retained higher β-sheet and lower α-helix content than *R. oryzae*, suggesting divergent proteolytic or structural modification pathways between strains.

### 3.4. Yield Stress

**Figure 4:**
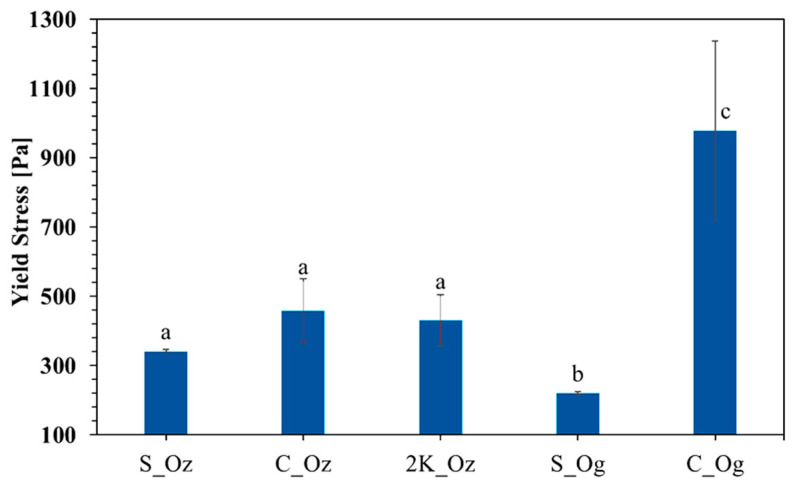
Yield stress (Pa) of fermented faba bean samples with different particle sizes and microbial strains after 48 h of fermentation. Samples include split, coarse, and 1000–2000 µm (2K) faba beans inoculated with *R. oryzae* (labelled as S_Oz, C_Oz, and 2K_Oz, respectively), and split and coarse faba beans inoculated with *R. oligosporus* (labelled as S_Og and C_Og, respectively). Data are presented as mean ± standard deviation. Different lowercase letters indicate statistically significant differences between samples (*p* < 0.05).

Yield stress was measured to assess the influence of particle size and microbial strain on rheological behaviour. No statistical differences (*p* > 0.05) were observed among samples fermented with *R. oryzae* (Figure 4). Specifically, S_Oz, C_Oz, and 2K_Oz exhibited yield stress values of 341.4 ± 6.3 Pa, 458.7 ± 92.1 Pa, and 430.6 ± 73.3 Pa, respectively. Interestingly, samples fermented with *R. oligosporus* showed significantly different yield stresses compared with *R. oryzae* (Figure 4): S_Og exhibited the lowest yield stress (219.9 ± 4.2 Pa; *p* < 0.05), whereas C_Og displayed the highest (978.9 ± 258.4 Pa; *p* < 0.05). These findings suggest that, while particle size alone may not significantly affect yield stress within the *R. oryzae* group, the choice of microorganism critically influences the rheological properties of fermented faba bean products.

### 3.5. Antioxidant and Antinutritional Analysis

**Figure 5:**
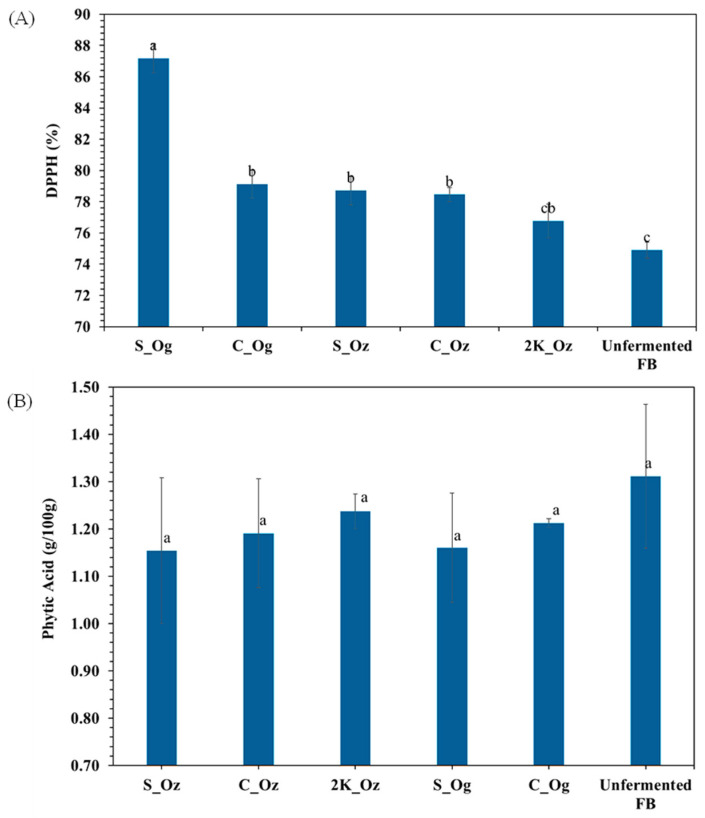
(**A**) DPPH radical scavenging activity (%) and (**B**) phytic acid content (g/100 g) of fermented faba bean samples with different particle sizes and microbial strains after 48 h of fermentation. Split, coarse, and 1000–2000 µm (2K) faba beans inoculated with *R. oryzae* are labelled as S_Oz, C_Oz, and 2K_Oz, respectively, while split and coarse faba beans inoculated with *R. oligosporus* are labelled as S_Og and C_Og, respectively. Data are presented as mean ± standard deviation. Different lowercase letters indicate statistically significant differences (*p* < 0.05) between samples.

Unfermented split faba bean showed 74.9 ± 0.5% DPPH radical inhibition. After 48 h of fermentation, all samples exhibited higher radical-scavenging activity (76.8–87.1%), depending on particle size (Figure 5A). No significant differences were observed among particle sizes (*p* > 0.05). Interestingly, no significant difference was observed between 2K_Oz and unfermented faba bean (*p* > 0.05). However, a significant difference in DPPH radical inhibition was found between S_Og and S_Oz, highlighting a combined effect of microbial species and particle size. Similarly, phytic acid content was not significantly (*p* > 0.05) altered across samples of varying particle sizes or microbial strains relative to the unfermented sample (Figure 5B). Phytic acid content in unfermented split faba bean was 1.31 ± 0.15 g/100 g, with only modest reductions after fermentation. Levels ranged from 1.15 to 1.24 g/100 g for *R. oryzae* treatments (S_Oz, C_Oz, 2K_Oz) and 1.16–1.21 g/100 g for *R. oligosporus* (S_Og, C_Og). Although fermentation enhanced antioxidant activity, it did not significantly reduce phytic acid under the tested conditions. Despite small changes, the consistent downward trend suggests partial phytate degradation, indicating some enzymatic activity by *Rhizopus* spp., albeit less pronounced than typically reported for lactic acid bacteria or phytase-rich fermentations.

## 4. Discussion

### 4.1. Effect of Particle Size and Rhizopus spp. on Microbial Growth

After 48 h of fermentation, distinct morphological characteristics were observed among the faba bean samples (Figure 1A–E), influenced by both particle size and microbial strain. Faba bean splits (S_Oz) and coarse particles (C_Oz) fermented with *R. oryzae* exhibited dense mycelial growth and uniform surface coverage. The S_Oz samples developed a mild sweet aroma, whereas C_Oz showed no perceptible odour. In contrast, the 2K_Oz samples displayed moderate fungal growth, a lighter surface colour, and lacked aroma, suggesting that the smaller particle size (1000–2000 µm) may have restricted oxygen diffusion and aeration, thereby creating suboptimal conditions for mycelial development [15]. Samples fermented with *R. oligosporus* (S_Og and C_Og) exhibited visibly weaker and more loosely bound mycelial growth. S_Og developed a darker appearance with a faintly sweet aroma, while C_Og was lighter in colour with a weak beany-to-sweet odour. These observations indicate that, even under identical fermentation conditions and substrate particle sizes, growth and sensory outcomes varied markedly between Rhizopus species, highlighting strain-dependent substrate interactions.

The strain-dependent physiological traits of the two fungi likely explain these differences. *R. oligosporus* produces shorter mycelia (150–400 µm), shows limited sporulation, and is adapted to lower oxygen conditions, resulting in reduced enzymatic activity. In contrast, *R. oryzae* forms longer mycelia (up to 1500 µm), exhibits stronger hydrolytic enzyme activity, and requires higher oxygen availability, enabling deeper substrate penetration and vigorous sporulation [35,36]. These physiological distinctions likely explain the observed variations in fungal development, surface morphology, and aroma profiles, reinforcing the importance of microbial selection in optimising solid-state fermentation.

### 4.2. Effect of Particle Size and Rhizopus spp. on Nutritional Properties

Fermented faba bean samples contained approximately 31.2–32.5% protein (Table 2), comparable to the unfermented control (31.3 ± 1.1%). These results align with previous studies [37,38], which reported minimal changes in protein content (29.7–30.9%) following fermentation with *Bacillus subtilis*, *Lactobacillus acidophilus*, or *Saccharomyces cerevisiae* after pre-treatments such as soaking, cooking, or autoclaving. Similarly, no statistically significant differences (*p* > 0.05) were observed in total carbohydrate or fat content, indicating that fermentation had little effect on overall macronutrient composition.

Although protein levels remained similar, SDS-PAGE analysis revealed distinct structural modifications caused by fermentation-induced proteolysis. Unfermented samples displayed protein bands between ~10 and 110 kDa, which shifted predominantly below 48 kDa after fermentation. Bands corresponding to convicilin disappeared, vicilin bands decreased in intensity, and legumin subunits remained largely intact (Figure 2). Increased intensity in the lower molecular weight region suggests the formation of smaller peptides. These findings are consistent with reports on faba bean flour fermented with A. oryzae and *R. oligosporus*, which showed the loss of bands at ~50–75 kDa and stronger bands at ~20 and 37 kDa [39]. This indicates active proteolysis, whereby fungal proteases hydrolyse complex proteins into smaller peptides altering molecular weight distribution [40].

Phytic acid content showed no significant difference between fermented and unfermented faba bean samples, consistent with previous findings [4]. In legumes, phytic acid degradation largely depends on phytase activity, derived either from the grain or the fermenting microorganisms. However, phytase activity is highly sensitive to pH, temperature, and fermentation time, and optimal conditions vary among plant species due to differences in endogenous and microbial enzyme activity [41,42]. Thus, optimising environmental parameters such as pH and temperature in future studies may enhance phytate degradation.

In contrast to phytic acid, antioxidant activity increased after fermentation, as reflected by enhanced DPPH radical scavenging (Figure 5). This suggests that microbial metabolism promoted the release or transformation of antioxidant compounds. These results agree with Toor, Kaur, Sahota and Kaur [43], who reported that *R. oligosporus* fermentation increased antioxidant activity by 76% in soybeans and 61% in chickpeas. After 48 h, split faba beans fermented with *R. oligosporus* (S_Og) exhibited higher antioxidant activity than *R. oryzae*-fermented samples (S_Oz), whereas no significant difference was observed in coarse particles (C_Og vs. C_Oz). This indicates that *R. oligosporus* may enhance antioxidant activity more effectively in finer particles, while *R. oryzae* produces a more uniform effect across particle sizes. Overall, microbial fermentation improved bioactive properties without substantially altering mineral-binding antinutrients under the tested conditions.

### 4.3. Effect of Particle Size and Rhizopus spp. on Secondary Structures

PCA of FTIR spectra revealed distinct separation among faba bean samples fermented with *R. oryzae* (S_Oz, C_Oz, and 2K_Oz), indicating particle size-dependent differences in secondary protein structure. Specifically, S_Oz (PC1^+^/PC2^−^) was associated with absorption bands at 1726 cm^−1^, 1840–1986 cm^−1^, and 2000–4000 cm^−1^, corresponding to protein amide I and lipid regions (Figure 3B,C). C_Oz (PC1^+^/PC2^+^) exhibited prominent wavenumbers in the 1700–2380 cm^−1^ and 3500–3950 cm^−1^ regions (Figure 3C; Supplementary Table S1), attributed to protein amide I and -OH groups, suggesting protein breakdown and peptide formation consistent with SDS-PAGE results (Figure 2). In contrast, 2K_Oz (PC1^+^/PC2^−^) showed peaks in the 700–873 cm^−1^ and 1313–1699 cm^−1^ ranges, linked to carbohydrate structures and protein secondary structures (amide II and III), indicating partial degradation of complex carbohydrates and conformational protein changes after fermentation.

Deconvolution of the protein amide I region further supports these observations, showing a reduction in β-sheet and α-helix content and a slight decrease in unordered structures across all fermented samples compared with the unfermented control (Table 3). Split samples fermented with *R. oryzae* and *R. oligosporus* (S_Oz, S_Og) clustered similarly (PC1^+^/PC2^−^), dominated by protein amide I and lipid bands. This suggests that the compact structure of larger particles restricted microbial access, resulting in limited fermentation effects. Coarse samples showed species-specific shifts: C_Og (PC1^−^/PC2^−^) correlated with protein amide II/III and carbohydrate regions, while C_Oz (PC1^+^/PC2^+^) aligned with amide I and -OH bands, indicating greater microbial activity and protein restructuring. *R. oligosporus* fermentation yielded higher β-sheet and lower α-helix content than *R. oryzae*, particularly in C_Og. Differences in β-turn and antiparallel β-sheet structures were also observed between strains. These patterns suggest that coarse particles, with their larger surface area and carbohydrate exposure, enhance strain-dependent protein rearrangements, as enzymatic activity and metabolite production vary between strains. Thus, particle size modulates substrate accessibility and diffusion, influencing the extent of protein restructuring [43,44]. The combined FTIR and SDS-PAGE data underscore the importance of both particle size and microbial strain in modulating protein conformation during fermentation. The observed reduction in ordered secondary structures (α-helix and β-sheet) suggests partial protein unfolding or breakdown, which can increase molecular flexibility and expose hydrophilic and hydrophobic regions. Such conformational changes are known to enhance solubility and digestibility, while promoting network formation and gelation through improved inter-protein interactions [45]. The partial proteolysis observed in SDS-PAGE further supports this interpretation, indicating that fermentation-driven structural rearrangements underpin the enhanced functional potential of faba bean proteins.

### 4.4. Unctional Properties

Structural firmness, expressed as yield stress, represents the minimum stress required to disrupt a material’s internal structure and initiate irreversible deformation. In food systems, it describes the resistance of protein, polysaccharide, or fat networks to breakdown, influencing perceived firmness and product stability. The yield stress measured after 48 h of fermentation reflects the structural integrity of the fermented faba bean matrix. Samples fermented with *R. oryzae* (S_Oz, C_Oz, and 2K_Oz) exhibited similar yield stress values, indicating that particle size had little influence on structural firmness under the tested conditions. This consistency suggests that *R. oryzae* effectively forms a dense, uniform network structure across particle sizes, as supported by morphological observations (Figure 1A–C), reflected in uniform mechanical resistance across samples.

In contrast, *R. oligosporus* exhibited particle size-dependent effects on yield stress. Split faba beans fermented with *R. oligosporus* (S_Og) showed markedly lower yield stress than those fermented with *R. oryzae* (S_Oz), indicating weaker network formation and lower viscoelastic resistance. Conversely, coarse faba beans fermented with *R. oligosporus* (C_Og) exhibited significantly higher yield stress than those fermented with *R. oryzae* (C_Oz), suggesting stronger microbial–substrate interactions and greater mechanical integrity.

These results demonstrate that yield stress is jointly determined by species-specific colonisation and substrate particle size, with *R. oligosporus* being more sensitive to surface exposure. This study provides the first evidence that microbial-driven network formation, combined with substrate particle size, critically influences the yield stress of fermented faba beans. Overall, both microbial strain and particle structure determine matrix cohesion and viscoelastic strength, highlighting the value of rheological assessment in optimising the textural properties of fermented faba bean matrices.

## 5. Conclusions

This study demonstrated that both faba bean particle size and *Rhizopus* species play decisive roles in shaping the nutritional, structural, and functional outcomes of solid-state fermentation. Particle size strongly influenced mycelial development and network formation, with coarse substrates supporting more uniform colonisation, particularly by *R. oryzae*. While total protein content remained stable, SDS-PAGE and FTIR analyses showed significant proteolysis and alterations in secondary protein structures, highlighting the importance of structural rather than compositional changes during fermentation. Fermentation enhanced antioxidant activity, most notably in split beans fermented with *R. oligosporus*. In contrast, phytic acid levels remained largely unchanged, suggesting limited phytase activity under the tested conditions. Rheological analysis further showed strain-dependent responses to particle size: *R. oryzae* consistently formed dense matrices across substrates, whereas *R. oligosporus* exhibited strong particle size dependence producing firm and cohesive networks in coarse beans but weak structures in split beans. Collectively, these findings indicate that no single substrate/microbe combination is universally optimal. Instead, fermentation outcomes can be tailored by matching particle size with microbial species, enabling targeted modulation of texture and nutritional attributes. This strain/substrate interplay offers a valuable strategy for designing legume-based fermented foods with desired functional qualities and establishes a foundation for optimising fermentation processes in future applications. Further work is recommended to directly quantify protein digestibility and solubility to substantiate the inferred functional improvements. Future studies should also apply robust biomass estimation methods (e.g., ergosterol analysis) to refine growth assessments and examine a broader spectrum of antinutritional factors including tannins, lectins, saponins, and α-galactoside oligosaccharides, which influence protein digestibility, mineral bioavailability, gastrointestinal tolerance, and overall consumer acceptance.

## Figures and Tables

**Table 1:** Procedure for preparing faba bean fractions of varying particle sizes.

Particle Size	Procedure
Split (S)	Used in original form (whole bean split in two halves, dried, and dehulled) with a non-homogeneous particle size distribution.
Coarse (C)	Split beans were manually broken into 3–4 pieces using a mortar and pestle, resulting in a non-homogeneous particle size distribution.
1000–2000 µm (2K)	Split beans were ground using a Kenwood mixer (Kenwood FDP65, London, UK) in pulse mode for 5–10 s with frequent pauses (total time 30 s), followed by sieving through mesh no. 18 (1000 µm) and 10 (2000 µm) to obtain a homogeneous particle size.

**Table 2:** Moisture content, pH, fungal growth, colour, and odour of faba bean samples of different particle size and fermented with *R. oryzae* and *R. oligosporus* after 48 h. Compositional analysis of freeze-dried fermented faba bean, including total carbohydrate (Total CHO), fat, protein, total dietary fibre (Total DF), and ash content, as a function of particle size and microorganism.

	Fresh Samples After 48 h of Fermentation					Freeze-Dried Samples After 48 h of Fermentation					
Sample ID	Moisture Content (%)	pH	Fungal Growth	Colour	Odour	Total CHO (%)	Fat Content (%)	Protein Content (%)	Total DF (%)	Dry Moisture Content (%)	Ash Content (%)
S_Oz	42.33 ± 0.96	7.2 ± 0.0	Dense, uniform, white mycelium network	Yellow brown	Mildly sweet	54.33 ± 3.65	0.75 ± 0.22	32.54 ± 0.19	8.92 ± 0.23	1.96 ± 0.05	2.70 ± 0.02
C_Oz	46.96 ± 1.03	7.2 ± 0.0	Dense, uniform, white mycelium network	Yellow brown	No	56.61 ± 1.57	0.65 ± 0.10	31.90 ± 0.66	8.64 ± 0.98	1.96 ± 0.05	2.82 ± 0.01
2K_Oz	41.58 ± 1.80	7.4 ± 0.0	Moderate, relatively uniform, white mycelium network	Yellow	No	55.10 ± 1.20	0.45 ± 0.15	31.71 ± 0.42	8.23 ± 0.75	1.87 ± 0.03	3.08 ± 0.02
S_Og	41.45 ± 1.54	7.4 ± 0.1	Slight, non-uniform, patches of mycelium	Brown	Mildly sweet	60.13 ± 2.56	1.19 ± 0.25	32.06 ± 0.70	10.20 ± 0.28	2.07 ± 0.06	2.71 ± 0.02
C_Og	44.15 ± 1.82	7.3 ± 0.1	Slight, non-uniform, patches of mycelium	Yellow brown	Lightly beany and mildly sweet	61.86 ± 6.06	1.06 ± 0.11	31.23 ± 0.25	10.55 ± 0.07	2.11 ± 0.03	2.87 ± 0.02

**Table 3:** Relative proportions of secondary structural components within the protein amide I region of fermented faba bean of different particle sizes and microorganisms after 48 h of fermentation. Faba bean split (S), coarse (C), and 1000–2000 µm (2K) samples were inoculated with *R. oryzae*, whereas split (S) and coarse (C) samples were inoculated with *R. oligosporus*.

	Secondary Structure Component/Wavenumber (cm^−1^)					
	β-Sheets [%]	Unordered Sheets [%]	α-Helices [%]	β-Turns [%]	Antiparallel β-Sheets [%]	β-Type [%]
Sample ID	1600–1638	1638–1650	1650–1660	1660–1680	1680–1688	1690–1695
S_Unfermented	5.16	3.92	1.91	1.68	0.77	1.43
S_Oz	2.61	1.76	0.85	0.85	0.72	0.61
C_Oz	2.86	2.00	1.14	0.95	0.76	0.94
2K_Oz	2.75	1.98	0.73	0.93	0.19	1.11
S_Og	2.99	1.99	0.87	0.86	0.58	0.69
C_Og	3.47	2.64	0.40	0.64	0.27	0.70

## Data Availability

The original contributions presented in the study are included in the article/Supplementary Materials, further inquiries can be directed to the corresponding authors.

## Supplementary Materials

The following supporting information can be downloaded at: https://www.mdpi.com/article/10.3390/foods14234105/s1, Figure S1: The moisture content (%) (A) and pH value (B) as a function of time of the fermented faba bean at different particle sizes and *Rhizopus* species; Table S1: Estimated wavenumber ranges in each quadrant of the loading plot; Figure S2: The FTIR spectra (3500–500 cm^−1^) of the unfermented faba bean split.

## Abbreviations

The following abbreviations are used in this manuscript:

2K	1000–2000 µm faba bean particle fraction
2K_Oz	1000–2000 µm faba beans inoculated with *R. oryzae*
ANOVA	Analysis of variance
ATR	Attenuated total reflectance
CAZymes	Carbohydrate-active enzymes
C	Coarse faba bean particle fraction
C_Og	Coarse faba beans inoculated with *R. oligosporus*
C_Oz	Coarse faba beans inoculated with *R. oryzae*
DF	Dietary fibre
DLATGS	Deuterated L-alanine-doped triglycine sulphate
DPPH	2,2-Diphenyl-1-picrylhydrazyl
FB_2K_Oz	1000–2000 µm faba beans inoculated with *R. oryzae*
FB_C_Og	Coarse faba beans inoculated with *R. oligosporus*
FB_C_Oz	Coarse faba beans inoculated with *R. oryzae*
FB_S_Og	Split faba beans inoculated with *R. oligosporus*
FB_S_Oz	Split faba beans inoculated with *R. oryzae*
FTIR	Fourier transform infrared spectroscopy
G′	Storage modulus
G6PD	Glucose-6-phosphate dehydrogenase
IR	Infrared
PCA	Principal component analysis
PP	Parallel plate (rheometer geometry)
PP25/P2	Parallel plate 25 mm/serrated plate geometry
rpm	Revolutions per minute
RT	Room temperature
S	Split faba bean particle fraction
S_Og	Split faba beans inoculated with *R. oligosporus*
S_Oz	Split faba beans inoculated with *R. oryzae*
SDS-PAGE	Sodium dodecyl sulphate–polyacrylamide gel electrophoresis
UV	Ultraviolet
