# Supplementary material for: Structure and Functionality of Fermented Faba Bean: Influence of Particle Size and Rhizopus spp
Source: Foods. 2025 Nov 29;14(23):4105. doi: 10.3390/foods14234105 (PMC12692153; doi:10.3390/foods14234105)
Supplement: Supplementary file 1 [file foods-14-04105-s001.zip › foods-3973662-supplementary.pdf]

Structure and Functionality of Fermented Faba Bean: Influence of Particle Size and *Rhizopus spp*.

Deepa Agarwal[1,2]*, Priyanka Kharangarh [1,2], Pengfei (Alfie) Hao[1,2], Mark I Bradbury[1,3], Pankaj Maharjan[4,5], Yakindra Timilsena[1,2], Cassandra K Walker[4,5], Monika S. Doblin[1,2], Roman Buckow[1,2]**

[1] *La Trobe Institute for Sustainable Agriculture and Food, La Trobe University, Bundoora, Melbourne, Victoria, Australia.*

[2] *School of Agriculture, Biomedicine and Environment, Department of Ecological, Plant and Animal Science, La Trobe University, Bundoora, Victoria, Australia.*

[3] *School of Allied Health, Human Services and Sport, La Trobe University, Bundoora, Victoria, Australia.*

[4] *Agriculture Victoria Research, Horsham SmartFarm, Horsham, Victoria, Australia.*

[5] *School of Applied Systems Biology, La Trobe University, Bundoora, Victoria, Australia.*

*Co-corresponding authors: D.Agarwal@latrobe.edu.au, R.Buckow@latrobe.edu.au

Supplementary Data

(A)

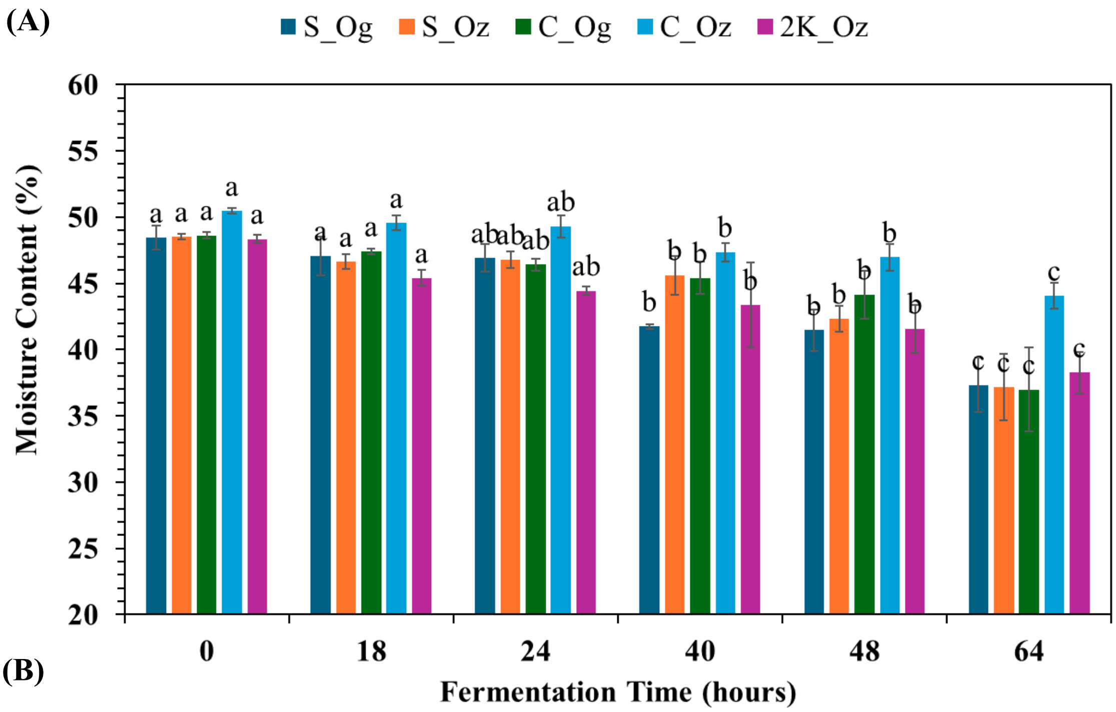

(B)

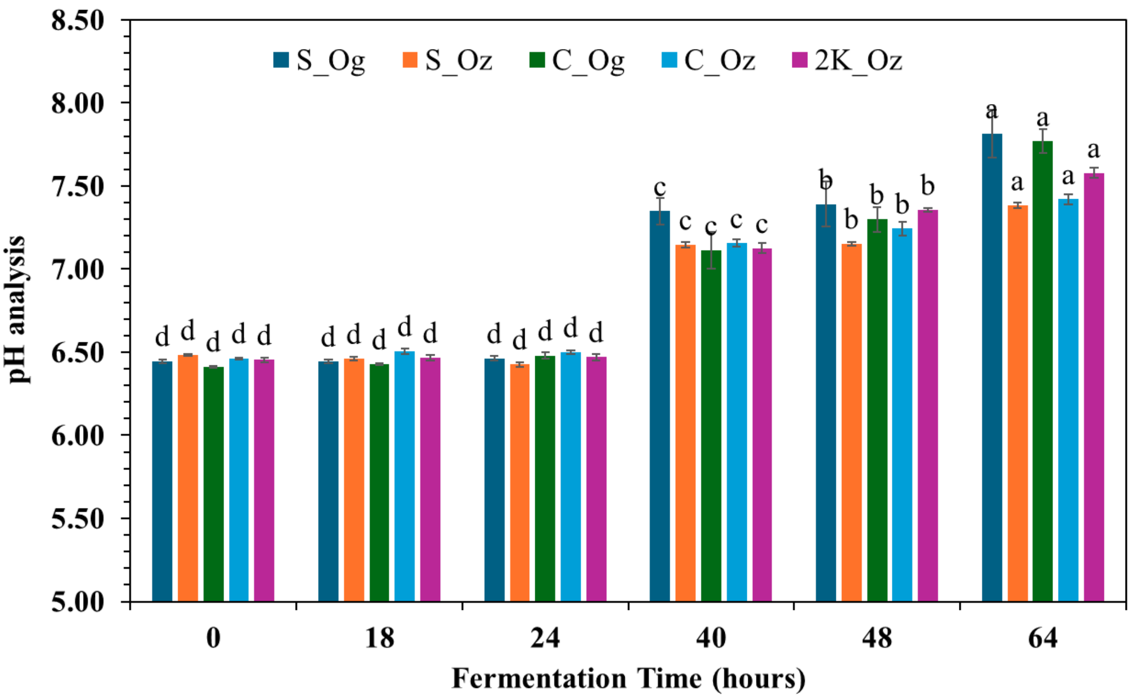

Figure S1: The moisture content (%) (A) and pH value (B) as a function of time of the fermented faba bean at different particle sizes and *Rhizopus* species. Split, coarse, and 1000–2000 μm faba beans, inoculated with *R. oryzae* are labelled as S_Oz, C_Oz, and 2K_Oz, respectively, whereas split and coarse faba beans inoculated with *R. oligosporus* are labelled as S_Og and C_Og, respectively. Data are shown as mean ± standard deviation, with different lowercase letters indicating significant differences ($p < 0.05$) as a function of time.

Table S1: *Estimated wavenumber ranges in each quadrant of the loading plot.*

PC1-/PC2+ (Top left)	PC1+/PC2+ (Top right)	PC1-/PC1 (Bottom left)	PC1+/PC2- (Bottom right)
399–600	1700–2380	451–1444	1726
780–873	3500–3950	1581–1658	1840–1986
1313–1699			2000–4000

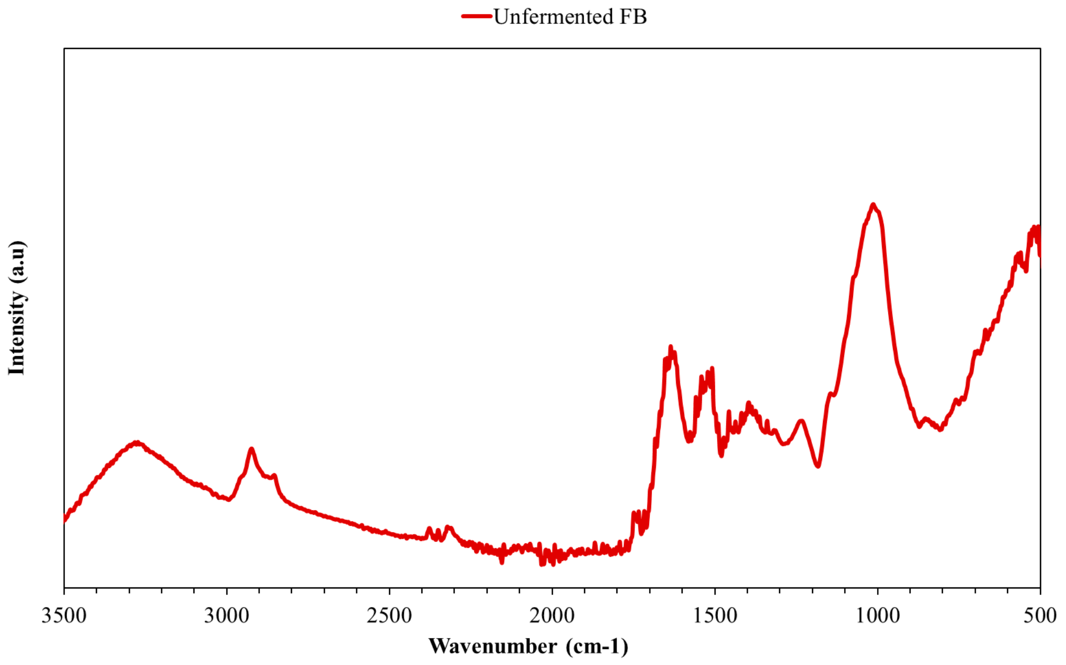

Figure S2: The FTIR spectra (3500-500 cm^{-1}) of the unfermented faba bean split.
